# Comments to Metallothionein as an Anti-Inflammatory Mediator

**DOI:** 10.1155/2009/426214

**Published:** 2009-10-12

**Authors:** Yong-Song Guan

**Affiliations:** Department of Oncology, State Key Laboratory of Biotherapy, West China Hospital of Sichuan University, P.O. Box 610041, Guoxuexiang Street 37, 61004 Chengdu, Sichuan, China

Dear Editor,

This paper introduces MTs as metalloproteins and their role in various inflammatory diseases. In the conclusion section, the authors state that MT-induction/enhancement and/or zinc supplementation to induce/enhance MT might be possible therapeutic options for inflammatory diseases [1]. 

To be an appropriate therapy on the level of genetics or molecule, the option should be well meditated because the targeting network for a cellular process is so complicated. First do no harm, which is the foremost principle for a therapy [2]. However, there is scarce any medical good without its concomitant harm. For example, it is reported that antibiotic treatment of bacterial infection, even if being successful, hinders development of acquired immunity so that the immune responses of the host confer no protection to secondary infection [3]. Anyway, the proposal for a therapeutic option needs discretion. 

Unfortunately, inflammation events and evidence provided in this paper are not sufficient enough to support MT as an anti-inflammatory mediator. Traumatic injury is not the mainstay of inflammatory diseases, and restrained inflammation is regarded as beneficial for host wound healing and defense toward infection [4]. Acute liver injury has quite different pathophysiology compared to viral hepatitis, liver cirrhosis, and liver cancer, which are chronic. Mechanism of anti-inflammatory mediation needs clearer presentation. 

Both MT and zinc are tightly regulated in the body. Zinc is crucial for the activation and binding of certain transcription factors through the participation in the zinc finger region of MT. Thus, MT regulates the level of zinc by binding and releasing this essential trace element. Too much zinc is also harmful to the body with resultant intoxication [5]. Surplus zinc is captured by MT the same as it detoxifies other heavy metals by forming inclusions and crystals [6, 7]. 

Simply inducing or enhancing MT is harmful because both increased and decreased expression of MT lead to malignant transformation of cells and ultimately cancer [8]. The former is found in several cancers including that of the breast, colon, and prostate, while the latter is detected in liver cancer [9]. In addition, higher levels of MT expression have proved to result in chemoresistance to drugs [10]. 

I would suggest fine tuning rather than sole induction or enhancement of MT, discussing one particular inflammatory disease instead of generalized ones, and investigating the effects of both increased and decreased MT expression with emphasis on the homeostasis of this mediator. In addition, zinc supplementation is indicated to zinc deficiency not to inflammation. 

*Yong-Song Guan* *Yong-Song Guan*
